# Neurovascular coupling in severe aortic valve stenosis

**DOI:** 10.1002/brb3.3155

**Published:** 2023-07-21

**Authors:** Ana Ovsenik, Matej Podbregar, Nikola Lakič, Martin Brešar, Pavle Boškoski, Ivan Verdenik, Andrej Fabjan

**Affiliations:** ^1^ Faculty of Medicine, Department of Biomedicine University of Ljubljana Ljubljana Slovenia; ^2^ Department of Cardiology University Medical Centre Ljubljana Ljubljana Slovenia; ^3^ Faculty of Medicine, Department of Internal Medicine University of Ljubljana Ljubljana Slovenia; ^4^ Department of Intensive Care General Hospital Celje Celje Slovenia; ^5^ Department of Cardiovascular Surgery University Medical Centre Ljubljana Ljubljana Slovenia; ^6^ Jožef Stefan International Postgraduate School Ljubljana Slovenia; ^7^ Department of Systems and Control Jožef Stefan Institute Ljubljana Slovenia; ^8^ Department of Obstetrics and Gynaecology, Division for Research University Medical Centre Ljubljana Ljubljana Slovenia; ^9^ Faculty of Medicine, Institute for Physiology University of Ljubljana Ljubljana Slovenia; ^10^ Department of Vascular Neurology and Neurological Intensive Care University Medical Centre Ljubljana Ljubljana Slovenia

**Keywords:** aortic stenosis, cerebral blood flow, neurovascular coupling, transcranial Doppler

## Abstract

**Objectives:**

Aortic stenosis (AS) is characterized by obstruction of blood outflow from the left ventricle, which can impair target organ perfusion such as the brain. We hypothesized that hemodynamic changes in AS may lead to dysfunction of cerebral blood flow regulatory mechanisms. The aim of our study was to evaluate neurovascular coupling in patients with AS by Transcranial Doppler ultrasonography.

**Methods:**

Neurovascular coupling was assessed using visually evoked cerebral blood flow velocity responses (VEFR) calculated as relative blood flow velocity changes in the posterior cerebral artery upon visual stimulation. We analyzed peak systolic, mean and end diastolic VEFR in 54 patients with severe AS and 43 controls in 10 consecutive cycles of visual stimulation. Repeated‐measures ANOVA test was used to compare cerebral hemodynamic data by group.

**Results:**

Patients with AS had significantly higher peak systolic (12.9% ± 5.6% and 10.5% ± 4.5%; *p* = .009) and mean VEFR (14.4% ± 5.8% and 12.2% ± 4.9%; *p* = .021) compared to controls, whereas only a tendency for higher end diastolic VEFR was observed (16.7% ± 6.9% and 14.4% ± 6.2%; *p* = .061).

**Conclusion:**

We have shown for the first time that patients with severe AS exhibit higher VEFR than controls indicating dysregulation of neurovascular coupling, which can be one of the factors contributing to development of cognitive decline.

## Graphical abstract

1. Patients with aortic stenosis exhibit higher visually evoked cerebral blood flow velocity responses than controls. 2. Left ventricular outflow obstruction in aortic stenosis may be associated with episodes of intermittent hypoperfusion, leading to dysregulation of neurovascular coupling. 3. Dysfunction in any of the cerebral blood flow regulatory mechanisms can cause functional deficits in the brain and precipitate cognitive decline.

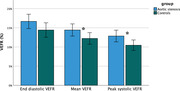

## 1 INTRODUCTION

Aortic stenosis (AS) is the most frequent primary valvular heart disease in the developed world, affecting 2.8% of population older than 75 years (Rashedi & Otto, 2015). Progressive aortic valve narrowing with concomitant left ventricular pressure overload and resultant left ventricular hypertrophy lead to the classic triad of AS symptoms including dyspnea, angina, and syncope (Joseph et al., 2017). In addition, cognitive scores of patients with severe AS are considerably lower compared with age, sex and education‐matched individuals without AS (Abdul‐Jawad Altisent et al., 2016). The precise mechanisms by which AS might precipitate brain dysfunction have not been fully elucidated, but may include improper initial upstroke time of velocity in carotid and vertebral arteries due to obstruction of blood outflow from the left ventricle (Kleczynski et al., 2017). In time, this can lead to dysfunction of cerebral blood flow (CBF) regulation, which may be reflected in cognitive decline (Ovsenik et al., 2021).

Multiple regulatory mechanisms overlap to provide tight CBF control due to the brain's high metabolic demand for oxygen, limited intracellular capacity for energy storage, rapid changes of metabolic demand with neuronal activity and enclosed cranium limited space (Ovsenik et al., 2021; Toth et al., 2017). Dysfunction in any of the regulatory mechanisms can cause functional deficits in the brain. Impairment of neurovascular coupling has been suggested as a major contributor to cognitive decline in hypertension and Alzheimer's disease (Girouard & Iadecola, 2006). Neurovascular coupling presents a complex functional association between astrocytes, neurons and microvasculature, which enables regional CBF to adapt to local neuronal activity (Phillips et al., 2016). The increase in regional CBF might be initiated from the direct effect of neuronal activity on the microvasculature, but the broader regional increase in CBF results from conduction of vasodilatation and myogenic responses to the remote upstream blood vessels (Iadecola, 2017). Fortunately, these are accessible to insonation with Transcranial Doppler Ultrasonography (TCD), which enables noninvasive assessment of neurovascular coupling by measurement of visually evoked cerebral blood velocity responses (VEFR) reflecting relative blood flow velocity changes in the posterior cerebral artery (PCA) upon visual stimulation (Aaslid, 1987; Panczel et al., 1999).

In this study, we hypothesized that neurovascular coupling may be impaired in AS due to chronic hemodynamic changes in the cerebral circulation. As there has been no report on neurovascular coupling in AS, our aim was to evaluate VEFR in patients with AS using TCD.

## 2 MATERIALS AND METHODS

### 2.1 Participants

The study included 54 patients with severe AS (35 men, 19 women; mean age 70.7 ± 9.8 years) and 43 age‐ and sex‐matched controls without AS or other cardiac disease (30 men, 13 women; mean age 70.6 ± 10.9 years). The AS group consisted of patients with severe AS, who were admitted to the Department of Cardiovascular Surgery, University Medical Centre Ljubljana, for isolated aortic valve replacement surgery without concomitant intervention on the other valves, coronary arteries, the ascending aorta and carotid arteries. The diagnosis and indication for surgery were undertaken according to the latest ESC Guidelines on valvular heart disease (Vahanian et al., 2021). Community‐dwelling controls without known cardiac disease were recruited from general practices of the Primary Healthcare Centre Ljubljana after undergoing echocardiography to exclude AS. Exclusion criteria for patients as well as controls included age <18 years, ≥50% stenosis of the common or internal carotid artery as assessed by duplex sonography, history of ischemic stroke in the PCA territory, epilepsy, alcohol abuse, left ventricular ejection fraction less than 30%, noncorrectable vision and poor temporal acoustic window.

The experimental procedures for the study were approved by the National Ethical Committee of the Republic of Slovenia (No. 0120–97/2018) and followed the principles of the Helsinki Declaration. A written consent was obtained from all the participants. Participants' characteristics are presented in Table 1.

**TABLE 1:** Participants’ characteristics.

	Aortic stenosis	Controls	*p* Value
*n*	54	43	
Clinical characteristics			
Age (years)	70.7 (± 9.8)	70.6 (± 10.9)	.957
Sex (*n* women)	19 (35.2%)	13 (30.2%)	.384
BMI (kg/m2)	28.0 (± 4.5)	25.7 (± 3.3)	.007
Arterial hypertension (*n*)	45 (83.3%)	18 (41.9%)	<.001
Diabetes mellitus (*n*)	17 (31.5%)	5 (11.6%)	.017
Hyperlipidemia (*n*)	34 (63.0%)	25 (58.1%)	.391
Smoking (*n*)	5 (9.3%)	2 (4%)	.323
Medication intake (*n*)			
Beta‐blocker	28 (51.9%)	4 (9.3%)	<.001
ACEi	33 (61.1%)	13 (30.2%)	.002
ARB	6 (11.1%)	5 (11.6%)	.592
MRA	1 (1.9%)	0	.557
Ca^2+^ blockers	13 (24.1%)	5 (11.6%)	.095
Diuretics	23 (42.6%)	7 (16.3%)	.005
Statins	32 (59.3%)	14 (32.6%)	.008
Hemodynamic measurements			
MAP (mmHg)	93 (± 15)	113 (± 22)	<.001
Et‐CO_2_ (mmHg)	36 (± 4)	37 (± 4)	.141
HR (/min)	71 (± 10)	66 (± 10)	.035
Mean PCA (cm/s)	32.4 (± 7.8)	31.4 (± 6.0)	.557
Mean MCA (cm/s)	43.5 (± 9.5)	45.5 (± 11.1)	.356
LVEF	65 (± 9)	66 (± 3)	.418

BMI: body mass index, ACEi: angiotensin converting enzyme inhibitors, ARB: angiotensin II receptor blockers, MRA: mineralocorticoid receptor blockers, MAP: mean arterial pressure, Et‐CO_2_: end‐tidal CO_2_, HR: heart rate, mean PCA: mean velocity in posterior cerebral artery, mean MCA: mean velocity in medial cerebral artery, LVEF: left ventricular ejection fraction.

Numerical variables are presented as mean ± SD, while categorical variables are presented as absolute values and percentages.

### 2.2 Research protocol

TCD examination was performed in a dark, soundproof room with temperature of around 22°C. The examination protocol was thoroughly explained to the participants before the examination.

Participants were instructed breathe calmly, not to talk and not to move heads during the procedure, which was performed in the sitting position. They had 2 MHz Doppler probes (Delica‐9 series, SMT Medical) mounted on the head using a flexible plastic commercial head holder. Blood flow velocity (BFV) in P2 segment of the right posterior cerebral artery (PCA) and M1 segment of the medial cerebral artery (MCA) were recorded through the temporal bones. The M1 segment of the left MCA served as a reference for the control of the nonspecific effects of systemic factors. The strongest signal of the corresponding artery was found with adjustment of probes' position, depth and sample volume, as described elsewhere (Phillips et al., 2016; Willie et al., 2011). Participants were seated 1.5 m from the screen onto which the visual stimulus was projected. Visual stimulation was performed with the use of a checkerboard with 100% contrast between light and dark squares that appeared in the manner of the inverse‐pattern with the frequency of 1 Hz. The experiment contained 10 cycles, consisting of a 20‐s rest phase (phase OFF) during which the subject had their eyes closed and the screen was dark, and a 30‐s phase of visual stimulation during which the patient focused on the red cross in the middle of the checkerboard on the screen (phase ON). Patients’ BFV in the left MCA and right PCA, arterial blood pressure (BP) and breath‐by‐breath partial pressure of end‐tidal carbon dioxide (Et‐CO_2_) were measured continuously. BP was monitored in a noninvasive manner with the cuff on the right ring finger on the level of the right hip under the level of the heart (Finapress 2300 Blood pre, Ohmeda) and Et‐CO_2_ across the facial mask with the use of a capnometer (Oscaroxy capnometer, Datex).

### 2.3 Signal processing and data analysis

The transcranial Doppler signals were demodulated down to audible frequency range and sampled at 8.1 kHz. The cardiorespiratory signals were sampled at 5 kHz. The signals were synchronized by using external digital input that was triggered at the start of each measurement session. Individual ON/OFF cycles in the signals that were affected by artifacts, such as external noise disturbances that significantly decreased the signal‐to‐noise ratio and could potentially lead to erroneous outcomes, were excluded.

An envelope curve of the PCA and MCA signals was used for velocity analysis. To evaluate peak systolic and end diastolic velocities, the peaks and valleys within the signal were identified, whereas mean velocities were estimated according to the Equation (1):

(1) $$\mathrm{Mean}\;\mathrm{velocity}\;=\frac{\mathrm{Systolic}\;\mathrm{velocity}+\left(2\times \mathrm{Diastolic}\;\mathrm{velocity}\right)}{3}.$$

HR was obtained from times between peaks in the PCA velocities and the Et‐ CO_2_ values were determined by peaks in the CO_2_ signal. Mean values of BP, Et‐CO_2_, and HR were calculated for ON and OFF phases and averaged for the 10 consecutive cycles.

**FIGURE 1:**
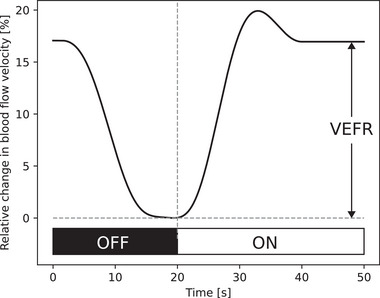
Schematic presentation of the visually evoked relative blood flow velocity time course in relation to baseline in PCA. VEFR: visually evoked cerebral blood flow velocity response, PCA: posterior cerebral artery.

According to the mechanism of neurovascular coupling, the stimulus leads to increase of blood flow velocity after approximately 1 s of visual stimulation, reaches the maximum after 5–10 s, followed by decrease and stabilization of the velocity on a lower level (plateau phase) (Aaslid, 1987). After removal of the visual stimulus, blood flow velocity falls to baseline level as presented schematically in Figure 1. For the assessment of neurovascular coupling, VEFR presenting the relative change of blood flow velocity in plateau phase in relation to baseline was calculated using the Equation (2):

(2) $$\mathrm{VEFR}\;=\frac{v\left(\mathrm{stimul}\right)-v\left(\mathrm{rest}\right)}{v\left(\mathrm{rest}\right)}\times 100\%,$$

where *v*(stimul) was average BFV during the last 10 s of visual stimulation and *v*(rest) average BFV during the last 5 s of rest (Boms et al., 2010; Fabjan et al., 2015; Olah et al., 2008; Rosengarten et al., 2001). VEFR were calculated separately for peak systolic, mean and end diastolic velocities of the PCA. Within one person, cerebral blood flow velocity data of 10 cycles were averaged and compared between the AS and control group (Fabjan et al., 2012). Additionally, relative PCA blood flow velocity changes in the early phase of visual stimulation were estimated for the time period 10 s from the peak onset in the hemodynamic response and compared between groups. Relative changes in the MCA BFV during stimulation were calculated according to the same formula as VEFR (Equation 2).

### 2.4 Statistical analysis

Statistical analysis was performed using IBM SPSS 27. Evaluation of normality was performed with Shapiro–Wilk's test. Normally distributed numerical variables were presented with arithmetic mean and standard deviation, while categorical variables were presented as absolute values and percentages (%). Statistical significance for intergroup differences was assessed by Student's *t*‐test for numerical variables and by Pearson's *χ* ^2^ test for categorical variables. Repeated‐measures ANOVA test was used to compare cerebral hemodynamic data by group. To evaluate for a possible habituation phenomenon, we performed multiple linear regression analysis on all cycles with cycle number as an independent variable. The limit of statistical significance was set at *p* < .05. Assuming a 5% SD for VEFR (Fabjan et al., 2015), a minimum of 40 patients per group was needed to detect a 3 % difference between groups at power of 80% and 5% alpha.

## 3 RESULTS

### 3.1 Baseline

Participants’ characteristics are presented in Table 1. Groups were matched in age and sex. Patients with AS exhibited higher BMI values, higher incidence of arterial hypertension, and diabetes mellitus and were more likely to take beta blockers, ACEi, diuretics, and statins. There were no statistically significant differences in baseline Et‐CO_2_, left ventricular ejection fraction (LVEF), mean MCA, and PCA velocities among the two groups; however, controls had higher MAP values and lower HR (Table 1).

### 3.2 Comparison of VEFR between groups

**FIGURE 2:**
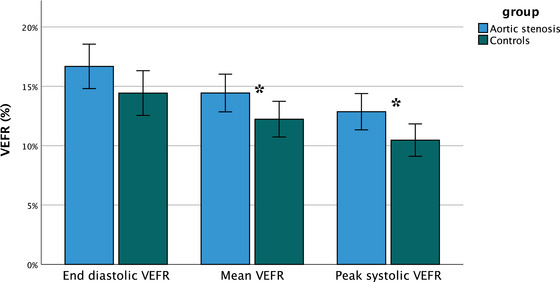
End diastolic VEFR, mean VEFR and peak systolic VEFR in both groups of participants presented as mean ± 2 SE. *Statistical significance computed from repeated‐measures ANOVA. VEFR: visually evoked cerebral blood flow velocity response.

Patients with AS had significantly higher peak systolic and mean VEFR compared to age‐ and sex‐matched controls, whereas only a tendency for higher end diastolic VEFR was found (*p* = .064) (Figure 2, Table 2). The most significant difference was observed in peak systolic VEFR (*p* = .009). As there was no difference in resting (phase OFF) PCA velocities between the groups, higher VEFR in AS group was contributed to higher PCA velocities during visual stimulation (phase ON) (Table S1). Moreover, higher relative PCA blood flow velocity changes were recorded in AS patients in the early phase of visual stimulation, however differences between groups did not reach statistical significance (Table S2).

**TABLE 2:** Relative cerebral blood flow velocity responses upon visual stimulation in patients with AS and controls.

	Aortic stenosis	Controls	*p* Value
Peak systolic VEFR (%)	12.9 (± 5.6)	10.5 (± 4.5)	.009*
Mean VEFR (%)	14.4 (± 5.8)	12.2 (± 4.9)	.021*
End diastolic VEFR (%)	16.7 (± 6.9)	14.4 (± 6.2)	.061
Δ Peak systolic MCA (%)	2.0 (± 2.4)	1.8 (± 2.4)	.775
Δ Mean MCA (%)	2.3 (± 2.4)	2.2 (± 2.8)	.987
Δ End diastolic MCA (%)	2.9 (± 3.4)	2.8 (± 3.9)	.971

AS: aortic stenosis, VEFR: visually evoked cerebral blood flow velocity response, PCA: posterior cerebral artery, MCA: medial cerebral artery.

Peak systolic, mean, and end diastolic VEFR represent the relative changes in PCA velocities upon visual stimulation, while Δ peak systolic, Δ mean, and Δ end diastolic MCA represent the relative changes in MCA velocities. Values are displayed as mean ± SD. *p* Values represent ANOVA test results. ^*^ *p* value < .05 is considered statistically significant.

**FIGURE 3:**
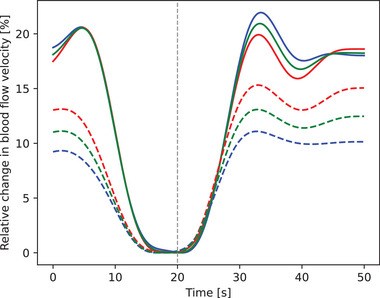
Visually evoked relative blood flow velocity time courses, averaged over 10 cycles for a sample subject from the aortic stenosis group (solid lines) and the control group (dashed lines). Blue lines represent peak systolic, green lines mean and red lines end diastolic values. The vertical dashed line at 20 s time represents the switch from OFF to the ON phase. PCA: posterior cerebral artery.

Average time trace of the peak systolic, mean and end diastolic PCA blood flow velocity response to the visual stimulus for a sample subject from the stenosis and control group is presented in Figure 3.

The relative MCA velocity changes were smaller compared to VEFR and were found to be no different between the AS group and control group (Table 2). During visual stimulation, MAP and Et‐CO_2_ did not change significantly between ON and OFF phases in both groups; however, there was a tendency for slightly higher HR during ON phase, which did not reach statistical significance (*p* = .066 and *p* = .051, respectively).

### 3.3 Variability of repeated VEFR

**FIGURE 4:**
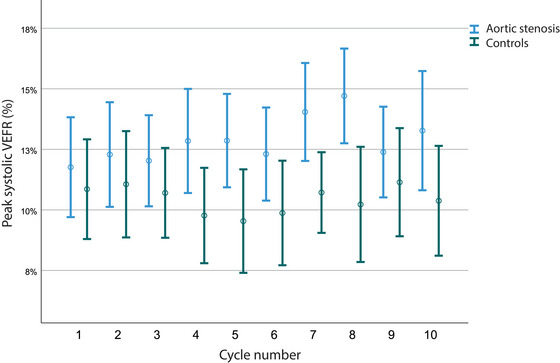
Peak systolic VEFR at each consecutive cycle in both groups of participants displayed as mean ± SD. VEFR: visually evoked cerebral blood flow velocity response.

Peak systolic VEFR at each consecutive cycle in both groups of participants are presented in Figure 4. As the differences in VEFR between groups do not answer the question, whether these can be contributed to different values of VEFR per se or to different trends of responses upon repetitive stimulation reflecting the habituation phenomenon, we performed multiple linear regression with VEFR as a dependent variable and cycle number and group as independent variables. Additionally, we added possible confounder variables (age, BMI, sex, smoking status) to obtain an adjusted model. There was no significant effect of cycle number to observed VEFR in none of the analyzed parameters. After adjustment for confounders, we obtained significant effect of group to peak systolic, mean and end diastolic VEFR (*p* < .001, Tables S3–S5).

## 4 DISCUSSION

To the best of our knowledge, this is the first study to evaluate visually evoked cerebral blood flow velocity responses (VEFR) as a measure of neurovascular coupling (NVC) in patients with severe aortic valve stenosis (AS) using Transcranial Doppler Ultrasonography (TCD). The main finding of this research is that patients with severe AS exhibit higher VEFR than controls which may indicate dysregulation of NVC.

NVC is a dynamic process constantly orchestrating the balance between local cerebral perfusion to its regional metabolic needs. As a crucial component of CBF homeostasis, disturbances in NVC have critical consequences on the brain function (Iadecola, 2017). Insufficient blood supply on one side of the spectrum or hyperperfusion on the other can disturb O_2_ delivery and be detrimental for neuronal function (Alosco et al., 2013; Rasmussen et al., 2015). The exaggerated hyperemic response can lead to an inappropriate increase in CBF in the metabolically active neuronal area, however at a cost of high energy expenditure, which is highly limited in the brain. The constant volume of intracranial blood volume implies that the cerebral blood overflow to active areas may lead to a shortage in vulnerable parts of the human brain, predisposing them to hypoperfusion. NVC dysregulation has been thoroughly studied in several pathological states such as traumatic brain injury, intracranial hemorrhage, migraine, cerebral ischemia and others (Fabjan et al., 2015; Hinzman et al., 2014; Koide et al., 2013; Rossetti et al., 2021; Zaletel et al., 2005). Furthermore, impairment of NVC has been suggested in various subgroups of patients with cardiac disease. A recent study in patients with atrial fibrillation reported on reduced cerebrovascular conductance upon visual stimulation when compared to age‐matched healthy controls (Junejo et al., 2020). Moreover, NVC response assessed by second‐order linear equation was found to be impaired in patients with heart failure with reduced ejection fraction (Aires et al., 2020). Even though, TCD was used in these two previous studies as well as in our study, the results might have differed due to diverse underlying cardiac pathology and data analysis.

Contrary to our expectations, patients with AS exhibited higher VEFR than controls. Episodes of intermittent cerebral hypoperfusion due to obstruction of blood outflow from the left ventricle are proposed as the main macro pathophysiological perturbation in aortic stenosis patients. This may in turn lead to structural and functional changes on the level of microcirculation, which is involved in NVC. Recent studies have shown that CBF measured by spin labeling MRI increased significantly after transcatheter aortic valve replacement, pointing out the possibility that CBF might indeed be diminished in severe AS patients (Vlastra et al., 2021). Reduced cerebral perfusion leads to hypoxia, which elicits a direct vascular effect and was shown to be associated with CBF changes (Willie et al., 2014). Some studies have revealed that hypoxia may lead to an increase in global CBF, while others have shown diminished regional CBF and reduction in the amplitude of NVC response during the hypoxic period (Ainslie & Subudhi, 2014; Lawley et al., 2017; Noth et al., 2008; Rossetti et al., 2021). How exactly intermittent hypoperfusion in AS affects NVC is not known yet. Our study was not designed to analyze individual components of NVC; however, an increased NVC response might in theory result from exaggerated hyperemic response to neuronal activity or increased neural excitability upon visual stimulation.

A possible mechanism in exaggerated hyperemic response could be increased sensitivity of endothelial cells to vasodilative stimuli. Various vasodilative molecules are believed to be involved in NVC such as nitric oxide, prostaglandins, epoxyeicosatrienoic acids, adenosine triphosphate, adenosine, H^+^, and K^+^ (Iadecola, 2017). An example of increased sensitivity of endothelial cells to vasodilative stimuli was presented in a study with familial migraine mice models, where increased hyperemic response was suggested to result from increased expression of inwardly rectifying potassium (Kir) channels on endothelial cells (Staehr et al., 2020). Kir channels promote K^+^ efflux and hyperpolarization of the cell membrane, which through various signaling pathways results in smooth muscle relaxation and vasodilatation (Knot et al., 1996; Longden & Nelson, 2015). Increased susceptibility of mice to vasospasm due to a genetic defect was believed to initially cause cerebral hypoperfusion and accumulation of metabolic factors, which in turn stimulated the increased expression of Kir channels on the endothelial blood cells and their sensitivity to vasodilative stimuli (Staehr et al., 2020). Similarly, episodes of chronic cerebral hypoperfusion in AS patients may lead to the accumulation of vasodilative substances and translational changes on the cellular level, which results in an exaggerated hyperemic response.

An alternative explanation for the higher VEFR could be increased neural excitability upon visual stimulation. The only electroencephalographic (EEG) study up to now analyzing neuronal activity in AS showed EEG patterns indicating changes in the cerebral circulation provoked by compression of carotid arteries. These changes were present even in patients who had no history of neurological symptoms pointing towards a latent vascular lesion which becomes manifest during temporary cerebral hypoxia (Prusik & Bazika, 1968). Furthermore, a study by Vecchio et al. (2012) revealed that acute ischemic hypoxia due to a cardiovascular or pulmonary crisis in chronic heart failure patients may induce EEG changes. Whether these EEG changes described in acute settings can be translated to the setting of chronic intermittent hypoperfusion remains unclear. However, there has been no study up to date studying evoked potentials as a measure of neuronal activation in AS. We believe that this mechanism is less likely to be the cause of increased VEFR.

Nevertheless, we hypothesized that there may be another reason for the differences in VEFR. As the comparison of peak systolic, mean and end diastolic VEFR between groups was performed using VEFR from 10 consecutive cycles, different trends (ascending or descending) in the repeated VEFR could have contributed to the final higher VEFR value in AS group. The succeeding decline in the evoked cerebral blood flow responses upon repetitive stimulation reflecting habituation phenomenon has been described in healthy subjects (Obrig et al., 2002). Habituation is considered to reflect an adaptive cortical mechanism protecting from sensory overstimulation (Thompson et al., 1979) and lactate accumulation (Sappey‐Marinier et al., 1992) as a response to sustained stimulus of equal intensity. A lack of habituation has been found in certain disease states which affect CBF regulation (Ambrosini et al., 2003). We used linear regression analysis, which showed no significant variability between the cycles in both groups that could support this hypothesis. Accordingly, lack of habituation is very unlikely to be the underlying explanation for higher VEFR in AS group.

Interestingly, there was no statistically significant difference in relative PCA blood flow velocity changes in the early phase of visual stimulation between groups, although the values tended to be higher in the AS group. The assessment of neurovascular coupling in our study was primarily based on the evaluation of the plateau phase of the visual blood flow velocity response in PCA (i.e., VEFR) to avoid the variability of the transitional phenomenon in the early phase. The method was found to be reliable and valid in previous literature (Boms et al., 2010; Fabjan et al., 2015; Fabjan et al., 2012; Olah et al., 2008), therefore the authors of this study used this methodology to answer the hypothesis according to the aim of our study.

The most prominent differences between groups were observed considering peak systolic VEFR compared to mean and end diastolic VEFR. Most studies use mean VEFR for blood flow velocity quantification, as peak systolic and end diastolic velocity indices describe the velocity extremes at two time points of one heart cycle, however they add valuable information on cerebrovascular hemodynamics. Functional TCD investigations demonstrated that the end diastolic blood flow velocity index may be more sensitive for changes in NVC mechanism than peak systolic velocity index, but is of no advantage if a stimulus is used that results in a clear blood flow velocity increase (Rosengarten et al., 2001), such as in case of visual stimulus used in this study. On the other hand, peak systolic velocity index is easier to obtain being less influenced by Doppler artifacts (Rosengarten et al., 2001). As it correlates with CBF in cardiac systole, these prominent differences between groups may be ascribed to peak systolic VEFR reflecting the pumping function of the heart, which is known to be disturbed in AS.

### 4.1 Limitations

Several potential limitations need to be considered. As TCD measures blood flow velocity rather than absolute CBF, the measurements of blood flow velocity are only proportional to CBF in case of constant diameter of the insonated vessel. This can only be assumed on a short‐term basis if the systemic parameters such as MAP, pH and EtCO_2_ remain constant. Transformation of the data to relative values improves correlation between velocity and blood flow changes as well as gains independence of the measurements from the insonation angle. Another limitation were the differences in baseline patient characteristics that could not be matched between groups. One of the differences was medication intake, which could have a potential effect on neural firing and resting vessel tone. Subanalysis of our data did not show any relevant effect of medication intake on VEFR estimates (Table S6), which is in accordance with previous research (van Rijssel et al., 2022). However, only limited conclusions can be withdrawn as the study was not designed to evaluate the effect of different medication subgroups on VEFR estimates. Nonetheless, we believe that dissimilarities in patients’ characteristics are not an important factor in the observed differences in VEFR between groups. Furthermore, we selected a homogenous group of patients with severe AS undergoing surgical aortic valve replacement, however, if these results can be generalized for the whole population of severe AS patients remains unknown.

## 5 CONCLUSION

This study shows higher visually evoked cerebral blood flow velocity responses in aortic stenosis patients compared to controls, which may indicate dysregulation of neurovascular coupling. Episodes of intermittent cerebral hypoperfusion due to obstruction of blood outflow from the left ventricle are proposed as the main macro pathophysiological perturbation in aortic stenosis leading to structural and functional changes on the level of microcirculation involved in neurovascular coupling. Future research including assessment of cerebral autoregulation, regulation of cerebral blood flow by gasses and autononomic nervous system control in aortic stenosis patients is mandatory to better elucidate how obstruction of blood outflow from the left ventricle influences cerebral blood flow regulatory mechanisms.

## AUTHOR CONTRIBUTIONS

All authors have made substantial contributions to the conception and design of the manuscript, acquisition of data, analysis and interpretation of data, drafting and critical revision of the article, and have approved the final version to be submitted.

## CONFLICT OF INTEREST STATEMENT

The authors declare no conflicts of interest.

## PATIENT CONSENT STATEMENT

A written consent was obtained from all the participants.

### PEER REVIEW

The peer review history for this article is available at https://publons.com/publon/10.1002/brb3.3155.

## Supporting information


**Table S1**. Peak systolic, mean and end diastolic PCA and MCA velocities in OFF and ON phases of visual stimulation. Values are displayed as mean ± SD. *p* values represent ANOVA test results. *p value < .05 is considered statistically significant. PCA: posterior cerebral artery, MCA: medial cerebral artery.
**Table S2**. Relative PCA blood flow velocity changes in the early phase of visual stimulation calculated for the time period 10 s from the peak onset. Values are displayed as mean ± SD, and *p* values represent ANOVA test results. *p* value < .05 is considered statistically significant. PCA: posterior cerebral artery.
**Table S3**. Multiple linear regression analysis with peak systolic VEFR as a dependent variable; cycle number and group as independent variables; sex, BMI, and smoking status as possible confounder variables.
**Table S4**. Multiple linear regression analysis with mean VEFR as a dependent variable; cycle number and group as independent variables; sex, BMI, and smoking status as possible confounder variables.
**Table S5**. Multiple linear regression analysis with end diastolic VEFR as a dependent variable; cycle number and group as independent variables; sex, BMI, and smoking status as possible confounder variables.
**Table S6**. The effect of medication on VEFR including all participants (aortic stenosis and control group). VEFR values are presented as mean ± SD. *p* values represent Student's *t*‐test results. *p value < .05 is considered statistically significant.

## Data Availability

The data that support the findings of this study are available from the corresponding author upon request.
