# Supplementary material for: Neurovascular coupling in severe aortic valve stenosis
Source: Brain Behav. 2023 Jul 21;13(8):e3155. doi: 10.1002/brb3.3155 (PMC10454277; doi:10.1002/brb3.3155)
Supplement: Supplementary file 1 — Table S1. Peak systolic, mean and end diastolic PCA and MCA velocities in OFF and ON phases of visual stimulation. Values are displayed as mean ± SD. p values represent ANOVA test results. *p value < .05 is considered statistically significant. PCA: posterior cerebral artery, MCA: medial cerebral artery. Table S2. Relative PCA blood flow velocity changes in the early phase of visual stimulation calculated for the time period 10 s from the peak onset. Values are displayed as mean ± SD, and p values represent ANOVA test results. p value < .05 is considered statistically significant. PCA: posterior cerebral artery. Table S3. Multiple linear regression analysis with peak systolic VEFR as a dependent variable; cycle number and group as independent variables; sex, BMI, and smoking status as possible confounder variables. Table S4. Multiple linear regression analysis with mean VEFR as a dependent variable; cycle number and group as independent variables; sex, BMI, and smoking status as possible confounder variables. Table S5. Multiple linear regression analysis with end diastolic VEFR as a dependent variable; cycle number and group as independent variables; sex, BMI, and smoking status as possible confounder variables. Table S6. The effect of medication on VEFR including all participants (aortic stenosis and control group). VEFR values are presented as mean ± SD. p values represent Student's t‐test results. *p value < .05 is considered statistically significant. [file BRB3-13-e3155-s001.docx]

**SUPPLEMENTAL MATERIAL**

|  | **Aortic stenosis** | **Controls** | ***p* value** |
| --- | --- | --- | --- |
| **OFF phase**  Peak systolic PCA (cm/s) | 52.7 (± 12.8) | 53.4 (± 10.7) | 0.758 |
| Mean PCA (cm/s) | 31.6 (± 7.7) | 33.2 (± 6.1) | 0.268 |
| End diastolic PCA (cm/s) | 21.1 (± 6.2) | 23.2 (± 4.5) | 0.073 |
| Peak systolic MCA (cm/s) | 75.8 (± 18.3) | 73.3 (± 15.4) | 0.428 |
| Mean MCA (cm/s) | 45.0 (± 10.2) | 45.2 (± 8.9) | 0.914 |
| End diastolic MCA (cm/s)  **ON phase**  Peak systolic PCA (cm/s)  Mean PCA (cm/s)  End diastolic PCA (cm/s)  Peak systolic MCA (cm/s)  Mean MCA (cm/s)  End diastolic MCA (cm/s) | 29.6 (± 7.8)  58.0 (± 14.5)  35.3 (± 8.8)  23.9 (± 6.9)  23.9 (± 6.9)  77.0 (± 18.5)  30.2 (± 8.0) | 31.1 (± 6.4)  57.2 (± 11.7)  36.3 (± 6.7)  25.9 (± 4.8)  74.3 (± 15.5)  46.0 (± 9.1)  31.8 (± 6.6) | 0.292  0.768  0.534  0.124  0.454  0.935  0.300 |

**Supplementary table 1.** Peak systolic, mean and end diastolic PCA and MCA velocities in OFF and ON phases of visual stimulation. Values are displayed as mean ± SD. p values represent ANOVA test results. *p value < 0.05 is considered statistically significant. PCA: Posterior Cerebral Artery, MCA: Medial Cerebral Artery.

|  | **Aortic stenosis** | **Controls** | ***p* value** |
| --- | --- | --- | --- |
| Δ Peak systolic PCA_early_ (%) | 14.4 (± 6.9) | 13.1 (± 5.1) | 0.321 |
| Δ Mean PCA_early_ (%) | 16.3 (± 6.4) | 14.1 (± 6.3) | 0.105 |
| Δ End diastolic PCA_early_ (%) | 18.4 (± 7.8) | 15.4 (± 9.2) | 0.087 |

**Supplementary table 2.** Relative PCA blood flow velocity changes in the early phase of visual stimulation calculated for the time period 10 s from the peak onset. Values are displayed as mean ± SD, p values represent ANOVA test results. p value < 0.05 is considered statistically significant. PCA: Posterior Cerebral Artery

| Model | | Coeff | | Std Coeff | t | Sig. | 95.0% CI for B | |
| --- | --- | --- | --- | --- | --- | --- | --- | --- |
|  |  | B | Std. Error | Beta |  |  | Lower Bound | Upper Bound |
|  | (Constant) | .222 | .033 |  | 6.655 | .000 | .156 | .287 |
|  | cycle number | .001 | .001 | .026 | .824 | .410 | -.001 | .003 |
|  | Aortic stenosis group | .025 | .007 | .122 | 3.619 | .000 | .038 | .011 |
|  | female sex | .010 | .007 | .045 | 1.342 | .180 | -.004 | .024 |
|  | BMI | -.001 | .001 | -.031 | -.920 | .358 | -.002 | .001 |
|  | smoker | .011 | .013 | .029 | .875 | .382 | -.014 | .036 |
|  | previous_smoker | -.012 | .008 | -.055 | -1.594 | .111 | -.027 | .003 |

**Supplementary table 3.** Multiple linear regression analysis with peak systolic VEFR as a dependent variable; cycle number and group as independent variables; sex, BMI and smoking status as possible confounder variables.

PCA: Posterior Cerebral Artery, VEFR: Visually Evoked Cerebral Blood Flow Velocity Response, BMI: Body Mass Index.

| Model | | Coeff | | Std Coeff | t | Sig. | 95.0% CI for B | |
| --- | --- | --- | --- | --- | --- | --- | --- | --- |
|  |  | B | Std. Error | Beta |  |  | Lower Bound | Upper Bound |
|  | (Constant) | .203 | .026 |  | 7.679 | .000 | .151 | .255 |
|  | cycle number | .001 | .001 | .032 | 1.008 | .314 | -.001 | .003 |
|  | Aortic stenosis group | .025 | .005 | .153 | 4.562 | .000 | .035 | .014 |
|  | female sex | .009 | .006 | .051 | 1.522 | .128 | -.003 | .020 |
|  | BMI | -.001 | .001 | -.045 | -1.350 | .177 | -.002 | .000 |
|  | smoker | .002 | .010 | .005 | .148 | .882 | -.018 | .021 |
|  | previous_smoker | -.006 | .006 | -.032 | -.923 | .356 | -.017 | .006 |

**Supplermentary table 4.** Multiple linear regression analysis with mean VEFR as a dependent variable; cycle number and group as independent variables; sex, BMI and smoking status as possible confounder variables.

PCA: Posterior Cerebral Artery, VEFR: Visually Evoked Cerebral Blood Flow Velocity Response, BMI: Body Mass Index.

| Model | | Coeff | | Std Coeff | t | Sig. | 95.0% CI for B | |
| --- | --- | --- | --- | --- | --- | --- | --- | --- |
|  |  | B | Std. Error | Beta |  |  | Lower Bound | Upper Bound |
|  | (Constant) | .222 | .033 |  | 6.655 | .000 | .156 | .287 |
|  | cycle number | .001 | .001 | .026 | .824 | .410 | -.001 | .003 |
|  | Aortic stenosis group | .025 | .007 | .122 | 3.619 | .000 | .038 | .011 |
|  | female sex | .010 | .007 | .045 | 1.342 | .180 | -.004 | .024 |
|  | BMI | -.001 | .001 | -.031 | -.920 | .358 | -.002 | .001 |
|  | smoker | .011 | .013 | .029 | .875 | .382 | -.014 | .036 |
|  | previous_smoker | -.012 | .008 | -.055 | -1.594 | .111 | -.027 | .003 |

**Supplementary table 5.** Multiple linear regression analysis with end diastolic VEFR as a dependent variable; cycle number and group as independent variables; sex, BMI and smoking status as possible confounder variables.

PCA: Posterior Cerebral Artery, VEFR: Visually Evoked Cerebral Blood Flow Velocity Response, BMI: Body Mass Index.

|  | **Aortic stenosis** | **Controls** | ***p* value** |
| --- | --- | --- | --- |
| **Peak systolic VEFR**  Beta blockers  ACEi  ARB  MRA  Ca^2+^  blockers  Diuretics  Statins  **Mean VEFR**  Beta blockers  ACEi  ARB  MRA  Ca^2+^  blockers  Diuretics  Statins  **End diastolic VEFR**  Beta blockers  ACEi  ARB  MRA  Ca^2+^  blockers  Diuretics  Statins | 0.118 (± 0.049)  0.118 (± 0.050)  0.117 (± 0.053)  0.118 (± 0.053)  0.120 (± 0.049)  0.117 (± 0.054)  0.112 (± 0.051)  0.136 (± 0.053)  0.133 (± 0.051)  0.133 (± 0.057)  0.135 (± 0.056)  0.136 (± 0.053)  0.134 (± 0.057)  0.129 (± 0.058)  0.159 (±0.066)  0.153 (± 0.057)  0.156 (± 0.068)  0.157 (± 0.067)  0.156 (± 0.063)  0.156 (± 0.067)  0.151 (± 0.074) | 0.119 (± 0.059)  0.118 (± 0.056)  0.127 (± 0.047)  0.093 (± .)  0.110 (± 0.065)  0.121 (± 0.050)  0.125 (± 0.054)  0.133 (± 0.060)  0.136 (± 0.060)  0.144 (± 0.044)  0.113 (± .)  0.130 (± 0.067)  0.137 (± 0.053)  0.141 (± 0.052)  0.153 (± 0.069)  0.161 (± 0.076)  0.166 (± 0.048)  0.141 (± .)  0.161 (± 0.081)  0.159 (± 0.066)  0.164 (± 0.056) | 0.912  0.957  0.563  0.632  0.485  0.727  0.226  0.821  0.782  0.545  0.691  0.725  0.818  0.299  0.701  0.591  0.614  0.809  0.784  0.801  0.329 |

**Supplementary table 6.** The effect of medication on VEFR including all participants (aortic stenosis and control group). VEFR values are presented as mean ± SD. p values represent Student t-test results. *p value < 0.05 is considered statistically significant.

VEFR: Visually Evoked Cerebral Blood Flow Velocity Response, ACEi: Angiotensin Converting Enzyme Inhibitors, ARB: Angiotensin II Receptor Blockers, MRA: Mineralocorticoid Receptor Blockers.
